# The Effect of Temperature and Milling Process on Steel Scale Utilized as a Pigment for Ceramic Glaze

**DOI:** 10.3390/ma13081814

**Published:** 2020-04-12

**Authors:** Hana Ovčačíková, Jozef Vlček, Vlastimil Matějka, Jan Juřica, Petra Maierová, Petr Mlčoch

**Affiliations:** 1 Department of Thermal Engineering, Faculty of Materials Science and Technology, VŠB-Technical University of Ostrava, 17. listopadu 2172/45, 738 01 Ostrava, Czech Republic; jozef.vlcek@vsb.cz (J.V.); petra.maierova@vsb.cz (P.M.); 2 Department of Chemistry, Faculty of Materials Science and Technology, VŠB-Technical University of Ostrava, 17. listopadu 2172/15, 738 01 Ostrava, Czech Republic; vlastimil.matejka@vsb.cz; 3 Department of Non-ferrous Metals, Refining and Recycling, Faculty of Materials Science and Technology, VŠB-Technical University of Ostrava, 17. listopadu 2172/15, 738 01 Ostrava, Czech Republic; jan.jurica@vsb.cz; 4 Třinecké železázárny, a.s, Průmyslová 1000 Staré Město, 739 61 Třinec, Czech Republic; petr.mlcoch@trz.cz

**Keywords:** scale, pigment, glaze, Fe_2_O_3_, milling

## Abstract

This study is focused on the evaluation of the re-utilizability of scale originated during the steel casting and steel rolling processes as a pigment for glazes. Non-oiled scale with Fe_3_O_4_ as the major phase were used as a coloring component of transparent glaze matrix in: (i) as received state, (ii) thermally pre-treated at 700 and 900 °C, (iii) mechanically treated in planetary ball mill (60, 120 and 240 min) and (iv) mechanically treated in vibratory disc mill (60 and 120 min). Prepared glazes were applied on the surface of ceramic tiles prepared from a commercially available white ceramic slurry. The resulting tiles with given glaze were thermally treated at 800, 900 and 1060 °C. The pigments were characterized by X-ray powder diffraction method (XRD), X-ray fluorescence spectroscopy (XRF), granulometry (PSD), thermogravimetric analysis (TG) and differential thermal analysis (DTA), scanning electron microscopy (SEM/EDAX). The color of the samples was described by the coordinates L*a*b* from CIELAB color space. The results showed that the non-oiled scale is suitable as the pigment for ceramic glazes. Careful control of the scale treatment process (mechanical as well as thermal) together with the temperature of final glaze firing is necessary to obtain the glaze of desired color and quality.

## 1. Introduction

**Figure 1:**
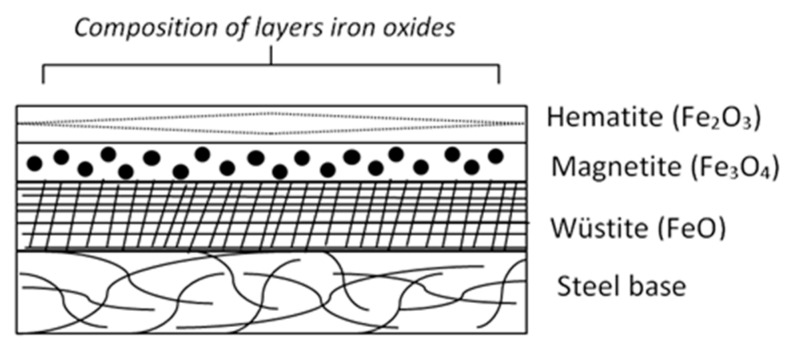
Layers of oxides. Adapted from [5].

Based on the statistics data, the global production of crude steel in 2018 reached approximately 1,808.6 million tons what means the increase of about 4.6% in comparison to 2017 (World Steel Association, 2017) [1]. During steel casting and rolling, a large quantity of mill scale originates and the estimated rate of the scale formation reaches approximately 2% from the given steel production [2]. As for the example reported, more than 1.4 million tons of mill scale are generated yearly in India and about 3.0 million tons are expected to be reached by 2020 [3]. Mill scale is iron oxide based waste material generated during the casting and rolling of steel. The dominant components of mill scale are oxides of iron (wüstite (FeO), hematite (Fe_2_O_3_) and magnetite (Fe_3_O_4_)). These oxides are generated mainly during the hot rolling of steel and are located on the outer surfaces of rolled plates, sheets or profiles [4]. The schematic representation of the composition of the oxide layer (scale) formed on the surface of steel is shown in Figure 1 [5].

Mill scale is very tempting industrial waste because of its richness in iron (about = 72% Fe). The major part of the mill scale is recycled in the place of its origin (steel production plants), the remaining part of the scale is available for the utilization in other industrial sectors or is stored on landfill.

The main characteristics of the pigments comprise the chemical composition, the optical and technological properties. Variety of the main pigment types include colored, colorless (under the specific temperature) or fluorescent materials. In general, the inorganic pigments are categorized into following groups: 1. white pigments, 2. black pigments, 3. colored pigments and 4. miscellaneous pigments (metal effect pigments, nacreous pigments, transparent pigment and luminescent pigment) [6]. The pigments could be organic or inorganic particulates that are usually insoluble and chemically stable in the medium in which they are dispersed. Thermal and chemical stability of the pigments are the next requirements.

The iron oxide pigments are widely used in paints, construction materials and coatings mainly due to their environmentally friendly character and chemical stability. The important properties with respect to the application of the iron oxides as the pigments include high tinting strength, hiding power and long durability [7]. There are two major processes used for the iron oxide pigment production. The first is based on the reaction of iron salts with alkalis [8], the second is so called Laux process based on the utilization of iron oxide during the nitro-benzene process [9].

Iron oxides could exist in seven crystalline phases, whereas the most common are α-Fe_2_O_3_ (hematite), γ-Fe_2_O_3_ (maghemite), Fe_3_O_4_ (magnetite) and Fe_1-x_O (wüstite); the less common are β- and ε-Fe_2_O_3_ phases and the low-temperature rhombohedrical structure of magnetite [10]. Hematite is the most stable iron oxide and it was traditionally used as a red pigment. Low price, low toxicity and high thermal and chemical stability predetermine the extensive utilization of the pigments based on α-Fe_2_O_3_ in coloring paints, plastics and enamels [11].

Despite the fact that the inorganic pigments should be evaluated comprehensively, the particle size as one of the parameter of the pigments has the strong effect on color of glaze. The particle size of the pigments for glaze application is usually reported between 1–20 μm and for the pigment with large sized particles the mechanical treatment by milling or crushing is recommended.

Other types of black pigments are based on the chromium, cobalt, manganese or nickel oxides [12,13,14,15]. Chromite is a relatively cheap natural pigment. Chromite can create brown spots but also change its color from yellow to brown. Another way to obtain iron oxide pigments is the use of selected waste materials with the suitable content of iron-bearing compounds (magnetite, goethite, hematite and maghemite). These pigments can be prepared through the isolation of given phase from waste, with or without subsequent chemical treatment [16,17,18,19].

The process of mechanical or mechanochemical treatment of Fe_2_O_3_ was used to alter the formation and phase transition of the iron oxides as reported for example in papers [20,21,22] and have been already utilized for the production of the iron oxide pigments. For example, nanosized Fe_3_O_4_ particles were prepared directly by milling of the metallic iron powder in distilled water using a planetary ball mill [23], FeO nanoparticles were fabricated via high-energy ball milling of the mixture of hematite and iron powders [24].

This paper describes the utilization of the steel scale as a pigment for the preparation of ceramic glazes. Scale treatment was divided in two experimental parts. In the Part 1, the scale was used in original and pre-calcined form, while in the Part 2 the scale was milled using two different mills (planetary ball and vibrational mill) without any thermal pre-treatment. The pigments were characterized by selected techniques of chemical and phase analysis. Particle size distribution (PSD) of the samples was determined using laser granulometry, thermogravimetric analysis (TG) and differential thermal analysis (DTA) was used to study the thermal stability of the scale. The morphology of the scale particles was studied using scanning electron microscopy equipped with energy dispersive microanalysis (SEM/EDAX). Scale was mixed with transparent glaze in a different weight ratios and applied on the surface of ceramic tiles. The ceramic tiles with colored glazes were fired at selected temperatures. The final glaze colors were described by L*a*b* values. The glazes with the commercial pigment based on α-Fe_2_O_3_ were prepared with the aim to understand if the glazes prepared with scale could reach the adequate quality.

## 2. Materials and Methods

### 2.1. Materials

Black colored scale (labeled as SC), was obtained from the Třinecké železárny a.s., Třinec, Czech Republic. Commercial white ceramic slurry for the preparation of the ceramic tiles was obtained from Pávek Keramika, spol. s r.o. (Doubravice nad Svitavou, Czech Republic). Commercially available transparent glaze powder (labeled as GL) was used as a glaze matrix and was obtained from company Glazura s.r.o. (Dobřín, Czech Republic). Commercially available iron pigment Fepren TP100 (Precheza a.s., Přerov, Czech Republic) (labeled as CP) was used for the selected experiments. Based on the available data sheet for Fepren TP100 this pigment contains more than 98 wt.% of α-Fe_2_O_3_.

The ceramic tiles with the dimensions 10 × 10 and 5 × 5 cm for the application of the glazes were prepared by casting of ceramic slurry in gypsum molds. After the unmolding, the tiles were dried at 105 °C to constant weight and further fired 6 h at 900 °C. The different types of glazes were prepared by 30 min long wet homogenization of the glaze matrix with studied admixture (scale or iron pigment) in a laboratory alumina ball mill (Brio Hranice s.r.o., Hranice, Czech Republic). The obtained suspension was mixed with water to reach the composition expressed by weight of glaze per liter in the interval 1.355–1.400 g∙L^−1^. The experimental work was further sub-divided into two parts based on the treatment of the scale used for the glaze preparation and the sample preparation processes are described in following Section 2.2 and Section 2.3.

### 2.2. Part 1: The Glazes with the Original Scale and Pre-Calcined Scale

Pre-calcination of the scale was performed by 6 h long calcination at 700 and 900 °C in a muffle furnace (LAC s.r.o., Židlochovice, Czech Republic). Based on the status of the scale treatment (original scale, scale calcined at 700 °C and scale calcined at 900 °C), three different glazes were prepared by 30 min long wet homogenization of powder glaze matrix with original and pre-calcined scale.

Using original scale, two glazes differing in weight portions of the scale −1 and 10 wt.% were prepared and spray coated on the surface of the ceramic tiles (10 × 10 cm) followed by the calcination at 1060 °C to provide the samples labeled GL+1SC/-/1060 and GL+10SC/-/1060 (see Table 1). Using pre-calcined scale, glazes with the amount of 10 wt.% of scale were prepared and coated by spraying on the surface of ceramic tiles (10 × 10 cm) to provide the samples GL+10SC/700/1060 (scale pre-calcined at 700 °C) and GL+10SC/900/1060 (scale pre-calcined at 900 °C), see Table 1. The amount of the glaze suspension sprayed on the surface of ceramic tiles was approximately 5 g. The sample GL+0SC/-/1060 (without scale) was prepared as well. The resulting samples were fired for 1 h at 1060 °C (heating rate 3 °C/min), after the heating the samples were allowed to cool down naturally in a furnace.

### 2.3. Part 2: The Glazes Prepared with the Scale Milled in Two Different Types of Mill Devices

In this part the original scale was pretreated by mechanical milling using vibratory or planetary mill (VM—vibratory disc mill; PM—planetary ball mill). Using the VM, the scale was grinded in a dry environment and the grinding time was set to 60 and 120 min (the samples were labeled as VM60 and VM120). Milling vessel in VM swings on a circle trajectory with frequency of 1200 rpm. Rate of the vessel filling was approximately 40%. Using the PM, the scale was grinded in ethanol and grinding time was 60, 120 and 240 min (the samples were labeled as PM60, PM120, PM240), the grinding speed was 300 rpm. After the milling the resulting slurry was dried at 105 °C.

The mixture of the glaze matrix and pretreated scale (5 or 10 wt.%) prepared by the procedure already described in Section 2.1. were applied on the surface of ceramic tiles (5 × 5 cm) by dipping method. The sample labels were assigned with the aim to indicate the type of milling device (VM, PM) and time of milling (0, 60, 120, 240 min), see Table 2.

The ceramic tiles covered with the glaze containing 5 and 10 wt.% of commercially available pigment labeled as 5CP and 10CP were prepared as well (note: the glazes with Fepren pigment were prepared in the same way as the glazes with scale, the procedure is described in Section 2.1.).

Resulting samples were heated up to the final temperature 800, 900 and 1060 °C for 6 h and stayed 1 h at the final temperature. After the heating, the samples were allowed to cool down naturally.

### 2.4. Characterization Methods

Chemical composition of the raw materials was determined using energy dispersive fluorescence spectrometer SPECTRO XEPOS (SPECTRO Analytical Instruments GmbH, Kleve, Germany), equipped with 50 Watt Pd X-ray tube. The powderized samples (4 g) were homogenized with a wax (0.9 g) and subsequently pressed into the pellets of diameter 32 mm using manual hydraulic press (Brio Hranice s.r.o., Hranice, Czech Republic) with the applied load 10 tons. Phase composition of the samples was characterized using a Bruker D8 Advance x-ray powder diffractometer (Bruker AXS GmbH, Karlsruhe, Germany). The diffraction patterns in the range of 5° to 70° 2θ were recorded under CoKα (λ = 1.78897 Å, U = 35 kV, I = 25 mA) radiation with scanning rate 2°/min using fast position sensitive detector VÅNTEC1. The registered diffraction patterns were evaluated using software EVA (Version 2, Bruker AXS GmbH, Karlsruhe, Germany) and the database PDF2 Release 2004 database (International Centre for Diffraction Data).

The morphology of the particles was characterized using the scanning electron microscope QUANTA 450 FEG (FEI, Hillsboro, OR, USA), the images were collected using secondary electron detector. The particle size distribution (PSD) of the scale was analyzed on the equipment Mastersizer (Malvern Panalytical Ltd., Malvern, UK). Measurements were performed in aquatic environment and ultrasound was used for homogenization of the suspension. The characterization of the thermal behavior of the scale was performed on TG/DCS analyzer STA504 (TA Instruments, New Castle, DE, USA). The sample of scale placed in alumina crucible was analyzed in temperature range 21 to 1100 °C in the dynamic atmosphere of N_2_ (5 L·h^−1^), the heating rate was 10 K·min^−1^. The reflectance spectra of the final glazes in the spectral range 400–700 nm were obtained using MiniScan EZ0828 spectrometer (HunterLab, Reston, VA, USA), model 45°/0°, viewing area small. The color of the glazes was expressed using CIE L*a*b* coordinates calculated for 10° observer and D65 illuminant. In CIE L*a*b* color space the L* is used to express the lightness of the color, a* is used for description of its position between magenta and green and b* is used for the description between yellow and blue [25,26]. The color of final glazes was also expressed using color-hex model used in HTML, CSS, SVG and other computing applications to represent colors [27,28] and further described using Munsell model in which the color is described using hue, lightness and chroma [29].

Milling and homogenization of the raw materials was performed using planetary ball mill type PM400 (Retsch, Haan, Germany) and vibratory disc mill BVM-2 (Brio Hranice s.r.o., Hranice, Czech Republic). The thermal treatment of the samples was performed using laboratory furnace LAC (LAC s.r.o., Židlochovice, Czech Republic).

## 3. Results and Discussion

### 3.1. Characterization of Raw Materials and Thermal Treatment of Scale

The chemical composition and loss on ignition values measured for scale (SC), transparent glaze (GL) and ceramic slurry is shown in Table 3.

**Figure 2:**
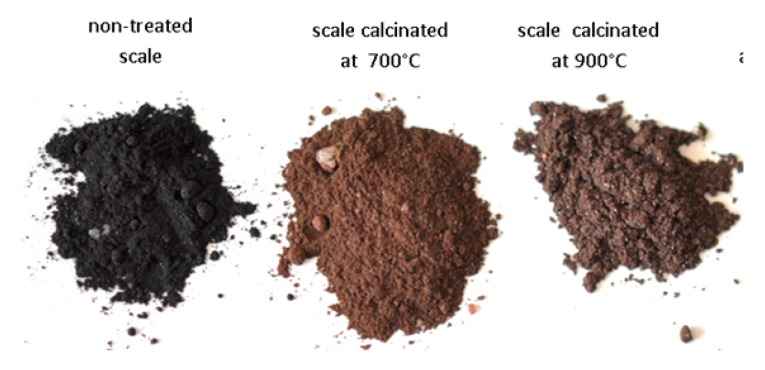
Images of original (non-treated) scale and scale calcined at 700 and 900 °C.

As expected, the principal chemical element composing the scale is the iron followed by silicon and calcium. Chemical composition of the ceramic slurry comprises silicon, aluminum, calcium, iron, magnesium, potassium and sodium (Table 3). The particle size distribution of the original SC sample ranged from 0.1–1000 µm with a maxima located at 15 μm. The images of the original SC and scale thermally treated at 700 and 900 °C are shown in Figure 2. Black color of the original scale imposes the presence of magnetite (Fe_3_O_4_) in this sample. Scale calcined at 700 °C is light brown in color and the scale calcined at 900 °C were transferred to dark brown as shown in Figure 2.

**Figure 3:**
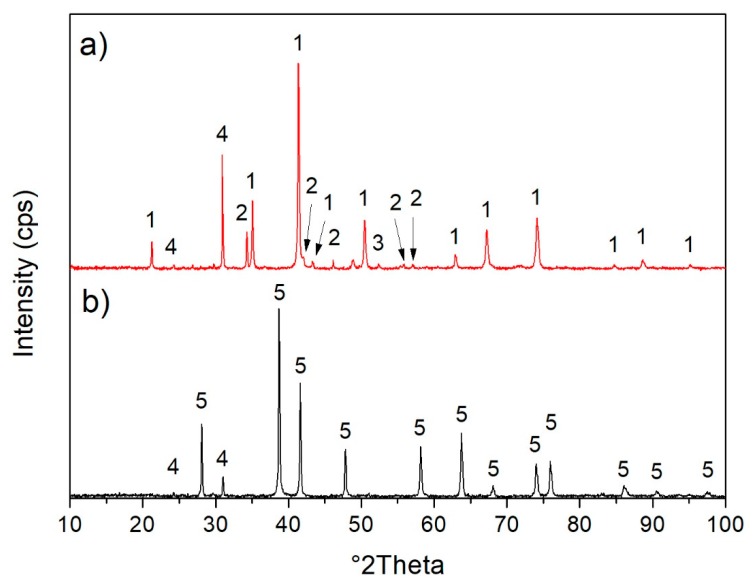
X-ray diffraction (XRD) patterns of (**a**) original scale; (**b**) scale calcined at 900 °C. (1—Fe_3_O_4_, 2—CaCO_3_, 3—FeO, 4—SiO_2_, 5—α-Fe_2_O_3_).

Based on the literature [20] the calcination of the phase Fe_3_O_4_ at about 400 °C leads to its transformation to γ-Fe_2_O_3_ and further heating above 500 °C causes the origination of α-Fe_2_O_3_. The variability of the color changes of Fe_3_O_4_ depends on its thermal treatment and comprises changes from black to brown color and even to orange color [20]. X-ray diffraction (XRD) patterns of the original scale and scale fired at 900 °C are presented in Figure 3. The presence of Fe_3_O_4_ phase in original scale was confirmed by XRD as shown in Figure 3a. The intensities of the diffraction peaks belonging to Fe_3_O_4_ phase are the most intensive what supports the fact that this phase is the main component of the studied original scale. Other phases identified in original scale are CaCO_3_, FeO and SiO_2_. Calcination of the original scale at 900 °C caused the phase transformation of Fe_3_O_4_ to α-Fe_2_O_3_ as documented by XRD pattern in Figure 3b. It is well known that during the heating of Fe_3_O_4_ in oxygen reach atmosphere the transformation of magnetite to γ-Fe_2_O_3_ (maghemite) or α-Fe_2_O_3_ (hematite) occurs. The oxidation sequences are described by Equations (1) and (2) [20]:

2Fe_3_O_4_ + 1/2O_2_ → 3γ−Fe_2_O_3_ (1)

2Fe_3_O_4_ + 1/2O_2_ → 3α−Fe_2_O_3_ (2)

The heating of the scale at 900 °C also caused the decomposition of CaCO_3_ as evidenced by the absence of the diffraction peaks belonging to the CaCO_3_ (Figure 3b). The unexpected increase in the intensity of SiO_2_ diffraction peaks after the calcination of scale (see Figure 3) could be attributed to the inhomogeneity of the original scale (the scale used for the calcination was not identical with the original sample used for XRD measurement). The other explanation is based on the hypothesis that there was an amorphous iron oxide in original scale, which transforms to the hematite during the calcination and thus the content of SiO_2_ proportionally decreased what is reflected in the decrease of the intensity of SiO_2_ diffraction peaks.

**Figure 4:**
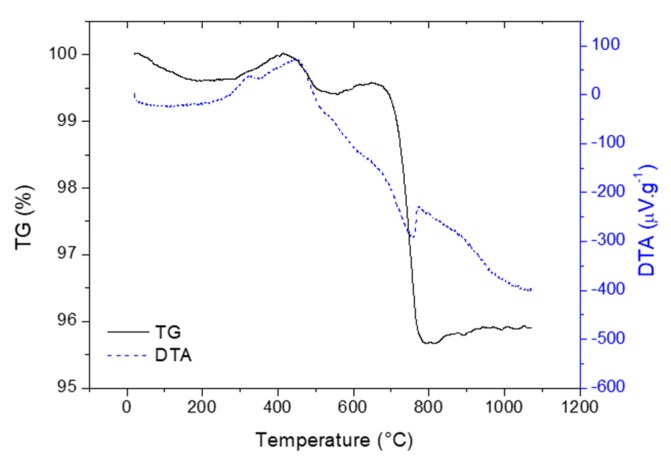
Thermogravimetric analysis (TG)/differential thermal analysis (DTA) curves registered for original scale.

TG/DTA curves registered for the original scale are shown in Figure 4. The decrease of mass in the temperature range 20–200 °C is mainly associated with the release of the physically absorbed water. The further decrease in sample weight in the temperature range 400–500 °C is probably connected to the decomposition of the iron hydroxides or iron hydroxide carbonate [30]. The presence of iron hydroxides as well as iron hydroxide carbonate was not proved by X-ray diffraction since these phases have the amorphous character. The endothermic peak in the temperature range 250–425 °C is attributed to the oxidation of FeO to Fe_3_O_4_ and Fe_2_O_3_ [31]. The first exothermic peak was observed on DTA curve in temperature region 310–480 °C. As mentioned above, in this temperature region the main phase of the scale Fe_3_O_4_ transform to γ-Fe_2_O_3_. Above 500 °C, the DTA curve shows declining character with the presence of small arm slightly above 500 °C, which is ascribed to γ-Fe_2_O_3_ to α-Fe_2_O_3_ phase transformation. The significant decrease in mass (TG) is evidenced in the temperature region 700–780 °C and the weight loss was approximately 4%. The endothermic peak with maxima at 750 °C is observable on the DTA curve and both the weight loss as well as the presence of the endothermic peaks signalize the decomposition of CaCO_3_ in this temperature region. The decomposition of CaCO_3_ was also confirmed by XRD method (Figure 3) as already mentioned in previous paragraph.

### 3.2. The Glazes Prepared with the Original Scale and Scale Fired at 700 and 900 °C

**Figure 5:**
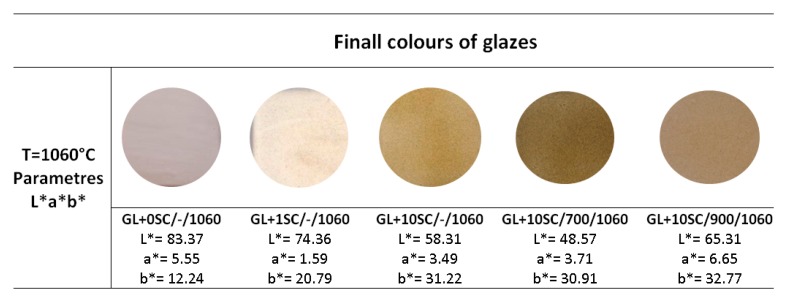
Images of the final glazes firing at 1060 °C and measured CIE L*a*b* coordinates.

The glazes: GL+0SC/-/1060 (0% scale, transparent glaze); GL+1SC/-/1060 (glaze with 1 wt.% of the original scale); GL+10SC/-/1060 (glaze with 10 wt.% of the original scale); GL+10SC/700/1060 (glaze with 10 wt.% of the scale pre-calcined at 700 °C) and GL+10SC/900/1060 (glaze with 10 wt.% of the scale pre-calcined at 900 °C) were applied on the surface of the ceramic tiles using spraying method and subsequently fired at 1060 °C. The visual comparison of the obtained samples is shown in Figure 5 together with the list of measured L*a*b* values.

Figure 5 shows the change of the tint of glazes. The changes of the color of ceramic tile with glaze without scale due to the due to the presence of non-calcined scale in two weight portions and due to the presence of 10 wt.% of scale calcined at 700 and 900 °C, respectively are clearly visible from Table 4. The originally greyish appearance of the glaze without scale turn to white appearance with addition of 1 wt.% of scale and further to the light brown if the content of the scale reached 10 wt.% (see Figure 5). Addition of the scale fired at 700 and 900 °C led to the dark brown appearance of the glaze (see Figure 5). In general, the color can be characterized by visual assessment or using a measuring technique. Colorimetry is a method used to describe a color accurately using trichromatic coordinates and the color of the object can be expressed for example in CIE L*a*b* color space. The L*a*b* values obtained for the studied glazes are listed in Figure 5. The CIELab coordinates (L*, a*, b*) were further converted to the HEX code [26]. Glaze GL+0SC/-/1060 has saturation 37.9% and lightness 79.8% and is defined by #DFCBB8 hexa code and is described as light grayish orange. The glaze GL+1SC/-/1060 has a saturation of 33.3% and a lightness of 67.6% and color is defined by #c8b591 hexa code and is described as a slightly desaturated orange. Similarly, the glaze GL+10SC/-/1060 has a saturation 31.2% and a lightness 48.4% with hexa code #A48855 and color is described as a dark desaturated orange, glaze GL+10SC/700/1060 has a saturation 36% and a lightness 38.6% with hexa code #8A703F and color is described as dark moderate orange. Glaze GL+10SC/900/1060 has a saturation 38.7% and a lightness 56.5% with hexa code #BD9964 and color is described as slightly desaturated orange. From a visual point of view, glaze GL+1SC/-/1060 is glossy transparent, GL+10SC/-/1060 became slightly colored and small dots appear on the surface of this glaze, glazes GL+10SC/700/1060 and GL+10SC/900/1060 have a more intensive light-yellow to brown-yellow color. The all prepared glazes (Figure 5) did not show any significant defects like blistering, crazing, pinholing and schivering what indicates that all the parameters of their preparation were optimally selected.

### 3.3. The Glazes Prepared with Grinded Scale

The reason for the mechanical treatment of the scale by milling process was to decrease the particle size. Two types of mills were used to modify the scale: vibratory disc mill (VM) and planetary ball mill (PM). Milling effectivity as described by the values d10, d50 and d90 obtained for every single milling is compared in Table 4.

**Figure 6:**
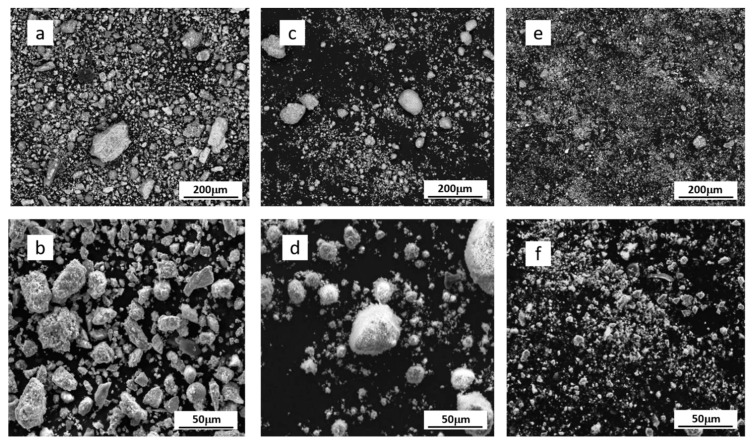
SEM images of (**a**,**b**) original scale; (**c**,**d**) scale VM120 and (**e**,**f**) scale PM120. The top images were acquired with lower magnification while the bottom images were acquired with higher magnification.

The milling process of the original scale (SC) decreased the size of its grains as evident from Table 4. In the case of VM, the 60 min long milling caused approximately 67% reduction of the d50 value of the particles of the original scale but further increase of milling time to 120 min caused slight increase in the d50 value as evident from Table 4. Longer milling for 120 min using VM led to the significant broadening of the particle size distribution of scale particles in comparison to 60 min long milling as evident from the data listed in Table 4. In the case of PM milling, the gradual increase of the milling time from 60–240 min had a slightly positive effect on the micronization of the scale particles mainly evidenced by the narrowing of the interval between d10 and d90 as shown in Table 4. The finest particles of scale were obtained using 240 min long micronization using PM. The original scale milled in VM120 and PM120 were studied using SEM technique and the respective images are shown in Figure 6.

The images shown in Figure 6a,c,e documented that the size and shape of grains of scale changed based on milling process. The particles of original non-pulverized scale are prismatic as shown in Figure 6a,b and it is evident that the single particles do not form the clusters. The SEM image obtained at higher magnification (Figure 6b) shows even the presence of spherical particles but their population in comparison with angular particles is negligible. SEM images of the particles of the scale milled using vibratory mill shown in Figure 6c,d, the images show the presence of prevailing part of grains with spherical shape. Any significant amount of angular grains was not observed in scale milled in vibratory mill. SEM images of the particles of the scale milled in planetary ball mill is shown in Figure 6e,f and demonstrates different particles obtained using this milling technique if compared with the original scales and with scales modified by vibratory mill. The milling in a planetary ball mill has a completely different effect than the milling in vibration mill and results in finer particles as evidenced by the values d90 in Table 4 and documented by SEM image of the particles in Figure 6e. Milling of the suspension of the scale with ethanol in planetary ball mill decreased size of grains but retained the shape of grain morphology as evident from comparison of the particles imaged in Figure 6b for original scale and Figure 6f for scale milled in planetary ball mill.

**Figure 7:**
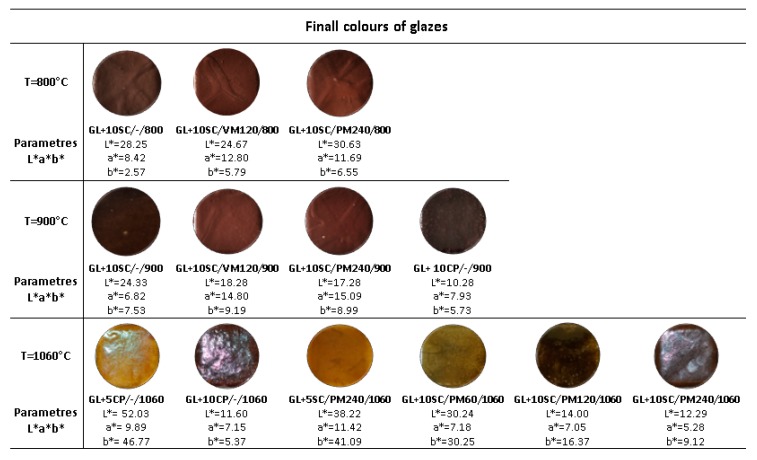
Image final glazes with scale and commercial pigment.

The effect of the grinding technique, time of grinding and the temperature of the thermal treatment of the glazes is documented by the appearance of the surface of the prepared samples in Figure 7, the quantitative description of the colors by L*a*b* coordinates is listed also in Figure 7.

Comparison of the glazes fired at 800 °C indicates similar color appearance for the samples with mechanically activated scales regardless the used milling technique, the same situation is for the glaze fired at 900 °C (Figure 7). The samples GL+10SC/VM120/800 and GL+10SC/VM120/900 exhibit very similar colors and are defined as very dark desaturated red. Surface of all the glazes fired at 800 °C have a matte appearance while at 900 °C the matte surface change to glossy (Figure 7). The glaze GL+10SC/PM240/800 calcined at 800 °C is of very dark grayish red color and little defect results that the glaze appears in a matt, the same glaze GL+10SC/VM240/900 treated at 900 °C appears as a glossy very dark desaturated red. Unevenness appeared on the surface of the samples GL+10SC/-/800, GL+10SC/VM120/800, GL+10SC/PM240/800 fired at 800 and 900 °C, GL+10SC/-/900, GL+10SC/VM120/900, GL+10SC/PM240/900.

Different defects occur on the surface of the glazes depending on the firing temperature. Small pinholes occurred at the surface of the glaze GL+10SC/-/800 fired at 800 °C. The reason for this defect is the presence of the dust on the glaze surface or release of the bubbles that have not healed over due to the gas release during the firing process. Fine cracks resulting from shrinkage differences between the clay body and glaze are another kind of the defects occurring on the surface of the final samples, e.g., GL+10SC/VM120/800.

The glazes prepared with the scale activated by milling in planetary ball mill (PM) and glazes prepared using commercially available iron pigment Fepren (samples assigned GL+10CP/-/900, GL+5CP/-/1060 and GL+10CP/-/1060) were also compared in Figure 7. Both the scale milled for 240 min and Fepren were dosed in the amounts 5 and 10 wt.% (samples GL+5CP/-/1060, GL+10CP/-/1060, GL+5SC/PM240/1060 and GL+10SC/PM240/1060) and it is evident that the color of the glazes differs significantly with the content of the pigment. The light yellow color was achieved for both GL+5SC/PM240/1060 and GL+5CP/-/1060 (the samples with 5 wt.% of the scale and commercial iron pigment) fired at 1060 °C while very dark glossy red-brow color was achieved for both GL+10SC/PM240/1060 and GL+10CP/-/1060 (the samples with 10 wt.% of the scale and commercial iron pigment). This fact proved the significant effect of the dosage of the scale and pigment on the final color appearance of the glaze.

In addition, the effect of the time of the milling of the scale (60, 120 and 240 min) on the color appearance of the glazes with 10 wt.% of the scale was investigated as well (samples GL+10SC/PM60/1060, GL+10SC/PM120/1060 and GL+10SC/PM240/1060 in Figure 7). The extension of the milling time significantly affected the color appearance of the final glaze as evident from the images shown in Figure 7. The shortest time of scale milling (60 min) led to origination of the yellow-green color of the final glaze, while the extension of the milling to 120 min led to the formation of the dark grey-brown glaze. Finally, the prolongation of the milling to 240 min led to the formation of highly glossy dark brown-black glaze. Based on the literature if the particles size of iron oxides is under 0.1 μm resulting pigments are of high porosity [18]. The firing of the glazes at different temperature evidenced the significance of this parameter on the final appearance of the glaze, whereas the increase of the firing temperature from 800 to 900 °C changed the matte to glossy character of the final glaze. It is well known fact that the Fe^3+^ ions are very reactive in mixture with glaze, frits and ceramic body. The diffusion of the Fe^3+^ ions is considered as the desired effect which promotes the evenness of the colored glaze. On the other hand, hematite particles could be also encapsulated in amorphous SiO_2_ and therefore the diffusion of iron from hematite to glaze is suppressed as reported in previous work published by Mozaffari et al. [32]. The suppression of this diffusion leads to the protection of red color of the final glaze. The hematite pigment in the red color is stable up to temperature 1000 °C, above the temperature 1000 °C the red color changed as evidenced in Figure 7.

## 4. Conclusions

Scale material that originates during the steel production (hot rolling) represents an interesting alternative solution to traditional pigments for glazes, which could be called the “scale glazes”. The results obtained within this research can be summarized as follows:

Glazes with thermally treated scale applied with spraying technique:

- Mill scale pre-calcinated at different temperatures (700 and 900 °C) changes its original black color to brown, because of the phase transformation of Fe_3_O_4_ to Fe_2_O_3_.
- The glaze with thermally treated scale applied using spraying method and subsequently fired at 1060 °C did not show any visible defect.
- Different amount of thermally treated scale (1 and 10 wt.%) added to the commercial glaze had an influence on the glaze saturation.
- Glazes with mechanically treated scale applied by dipping:
- Milling for 60, 120 or 240 min reduced the size of the scale particles and changed the morphology of the particles.
- Different firing temperature (800, 900 and 1060 °C) of glaze containing the mechanically treated scale formed the glazes with various colors—dark red, brown color, yellow-brown.
- Firing of the glazes at the temperatures 800 and 900 °C led to the formation of defects (pinholing and crazing) on the surface of glaze, and the surface exhibited the matt character.
- Glazes prepared at the temperature 1060 °C were compact, without any evident defects and the surface had a glossy character.
- The glazes prepared with milled scale fired at 1060 °C reached similar quality as the glazes prepared with the commercial pigment.

The results show that using the different methods of scale pre-treatment and different methods of glaze application the scale glaze of different colors and appearance can be prepared. With tuning of the scale pre-treatment parameters, the scale glaze with the parameters similar to glazes with the commercial pigments could be obtained. The selection of the glaze application procedure and final firing temperature affect the presence of the defects on the surface of final glaze and thus the glaze of desired quality can be prepared. Although the milling of the scale increase the cost of the scale pigment, the positive results indicate that the mechanically treated scale could represent the alternative material to the traditional iron oxide pigments. The utilization of LCA and LCCA methods would be necessary to confirm whether the utilization of the steel scale as the pigments could be an effective way how to utilize the non-recyclable part of the scale.

## Figures and Tables

**Table 1:** Formulation of glazes with different amount of original scale and thermal treatment.

Samples	Amount of the Materials (wt.%)		Pre-Calcination Temperature
	Transparent Glaze (GL)	Scale (SC)	
GL+0SC/-/1060	100	0	−
GL+1SC/-/1060	99	1	−
GL+10SC/-/1060	90	10	−
GL+10SC/700/1060	90	10	700 °C
GL+10SC/900/1060	90	10	900 °C

Note: Explanation of labeling of the samples: GL+XSC/PT/FT. X—content of scale (wt.%), PT—scale pre-treatment temperature (– Means the scale was not thermally pretreated) and FT—firing temperature.

**Table 2:** Composition of glazes with milled scale and used commercial pigment.

Samples	Amount of the Materials (wt.%)			Temperature of Firing (°C)			Milling Time (min)
	Transparent Glaze (GL)	Scale (SC)	Fepren (CP)	800	900	1060	
GL+10SC/-/800	90	10		x	−	−	−
GL+10SC/-/900	90	10		−	x	−	−
GL+10SC/VM120/800	90	10		x	−	−	120
GL+10SC/VM120/900	90	10		−	x	−	120
GL+10SC/PM60/1060	90	10		−	−	x	60
GL+10SC/PM120/1060	90	10		−	−	x	120
GL+10SC/PM240/800	90	10		x	−	−	240
GL+10SC/PM240/900	90	10		−	x	−	240
GL+10SC/PM240/1060	90	10		−	−	x	240
GL+5SC/PM/240/1060	95	5		−	−	x	240
GL+5CP/-/1060	95	−	5	−	−	x	−
GL+10CP/-/900	90	−	10	−	x	−	−
GL+10CP/-/1060	90		10	−	−	x	−

Note: Explanation of labeling of the samples: GL+XSC/M/FT. X—Content of scale (wt.%), M—type and time of milling (PM—planetary ball mill, VM—vibratory mill, 60, 120, 240—time of milling in minutes) and FT—firing temperature.

**Table 3:** Chemical composition of raw materials.

Oxides	Amount of the Materials (wt.%)		
	Scale (SC)	Transparent Glaze (GL)	Ceramic Slurry
Na_2_O	0.42	5.5	1.22
MgO	0.78	<0.3	2.83
Al_2_O_3_	1.97	9.1	12.42
SiO_2_	10.2	80.1	69.57
P_2_O_5_	0.21	<0.01	<0.0012
SO_3_	0.58	0.12	0.19
K2O	0.40	0.88	2.08
CaO	6.3	0.30	4.52
TiO_2_	0.086	0.13	0.56
MnO	0.67	0.006	0.07
Fe_2_O_3_	73.6	0.22	4.09
BaO	−	0.054	0.05
Loss on ignition	4.2	1.40	1.4

**Table 4:** Granulometry of milled scale.

Samples	Type of Device	Milling Time (min)	Granulometry µm		
			d10	d50	d90
SC	−		1.78	13.20	64.80
VM60	vibratory disc mill	60	0.85	4.44	21.60
VM120		120	1.32	6.04	50.30
PM60	planetary ball mill	60	1.76	6.05	27.70
PM120		120	2.07	6.04	19.40
PM240		240	1.23	4.69	15.10
